# Clinical Usefulness and Cut-Off Value of Computed Tomography-Measured Visceral Adipose Tissue in Coronary Artery Disease

**DOI:** 10.3390/diagnostics16030483

**Published:** 2026-02-05

**Authors:** Yi-Jhen Hsieh, Tsyh-Jyi Hsieh, Chung-Han Ho, Kung-Hsun Weng, Yi-Chen Chou

**Affiliations:** 1 Department of Electrical Engineering, National Cheng Kung University, Tainan 701401, Taiwan; 2 Department of Medical Imaging, Chi Mei Medical Center, Yongkang, Tainan 710402, Taiwan; 3 Department of Radiology, Faculty of Medicine, College of Medicine, Kaohsiung Medical University, Kaohsiung 807378, Taiwan; 4 Department of Medicine Research, Chi Mei Medical Center, Yongkang, Tainan 710402, Taiwan; 5 Department of Information Management, Southern Taiwan University of Science and Technology, Tainan 710301, Taiwan

**Keywords:** computed tomography (CT), coronary artery disease (CAD), obesity, visceral adipose tissue (VAT), Taiwanese, coronary computed tomography angiography (CTA)

## Abstract

**Background/Objectives**: Abdominal obesity, especially visceral adipose tissue (VAT), is an independent risk factor for coronary artery disease. This study aimed to investigate the association between single-slice CT-measured VAT and significant coronary artery stenosis and to establish an optimal VAT cut-off value for Taiwanese adults. **Methods**: Patients who underwent abdominal CT and coronary CT angiography (CTA) within 1 month of each other were enrolled in this retrospective study. Axial images of abdominal CT at the L4 pedicle level were selected for further VAT, subcutaneous adipose tissue, and paraspinal muscles analysis. Significant coronary artery stenosis was defined as any luminal stenosis of >50% of the diameter of the vessel that was measured in coronary CTA. Anthropometric and laboratory measurements, including height, weight, waist circumference (WC), blood pressure, blood glucose, and blood lipids, were also analyzed. **Results**: A total of 779 patients (300 females; 54.9 ± 9.96 years) were enrolled. Only VAT and systolic blood pressure correlated significantly with significant coronary artery stenosis. No significant differences were found in other demographic and anthropometric characteristics between the groups with and without significant coronary artery stenosis. **Conclusions**: Single-slice CT-measured VAT was associated with significant coronary artery stenosis, and a lower VAT cut-off is recommended for the Taiwanese population.

## 1. Introduction

Coronary artery disease (CAD) remains a major cause of mortality in both developed and developing nations [1]. To effectively prevent CAD, the adoption of healthy lifestyle measures—including a balanced diet; regular physical activity; smoking cessation; and adequate management of established risk factors such as dyslipidemia, hyperglycemia, and hypertension—has been strongly advocated [2,3]. These risk factors of CVD are components of the metabolic syndrome, and obesity is widely recognized as a significant precursor to developing metabolic syndrome [4,5].

However, increasing evidence indicates that obesity, particularly central (abdominal) obesity, acts as an independent risk factor for CAD and represents a pivotal component of the metabolic syndrome [6,7,8]. Recent studies suggest that obesity is not merely a state of energy storage but rather a manifestation of adipose tissue dysfunction [4,5,9,10,11,12]. This dysfunction is a widely discussed phenomenon that disrupts adipokine secretion and is intimately associated with multiple diseases, including metabolic syndrome and CVD [4,10,11,12,13,14].

Among anthropometric indicators of obesity, body mass index (BMI) remains the most commonly used measure of overall adiposity; however, it is less specific. Waist circumference (WC), typically measured at the level of the iliac crest, is widely used to diagnose abdominal obesity and is considered a stronger predictor of CAD outcomes [7,8]. In addition to BMI, TG, and HDL-C measurements, WC is used to calculate the visceral adiposity index, which indirectly assesses visceral fat function [4]. Furthermore, several in-depth studies using computed tomography (CT) or magnetic resonance imaging (MRI) have demonstrated that the associations between abdominal fat and CAD differ according to specific patterns of fat distribution [15,16]. Although both visceral adipose tissue (VAT) and subcutaneous adipose tissue (SAT) correlate with metabolic risk factors for CAD, VAT has been shown to exert a more pronounced effect on atherogenesis and cardiovascular outcomes, serving as an independent marker of CAD-related morbidity and mortality [17,18]. A cut-off value of 100 cm^2^ for VAT is commonly recommended for identifying metabolic syndrome and CAD risk [19,20,21]. Nevertheless, recent studies conducted in Asia have proposed lower optimal cut-off values of 80–85 cm^2^ [9,22].

Despite these findings, few studies have evaluated the precise relationship between CT-quantified VAT and CAD within Taiwanese populations. Therefore, this study aimed to investigate the association between single-slice CT-measured VAT and significant coronary artery stenosis and to establish an optimal VAT cutoff value for Taiwanese adults.

## 2. Materials and Methods

### 2.1. Study Design and Sample

This retrospective study enrolled patients who underwent both abdominal CT and coronary CT angiography (CTA) at a single institution between January 2015 and October 2023. The source population was comprehensive, encompassing outpatients, inpatients, and participants in health screening programs. Key inclusion criteria were defined as follows:

• Presence of pre-enhanced abdominal CT images extending to the L4 vertebra level.
• Satisfactory imaging quality of coronary CTA, with clear vascular margins sufficient for measuring vascular stenoses.
• A temporal interval of less than one month between the abdominal CT and the coronary CTA examinations.

Given the inherent limitations of a retrospective study, obtaining visceral adipose tissue (VAT) data precisely on the day the coronary CTA was performed was largely infeasible. Consequently, we included cases in which abdominal CT was performed within 1 month of coronary CTA to minimize potential variation in patient condition between the two assessments and to ensure sufficient sample size.

Exclusion criteria comprised individuals with significant motion artifacts in their images, a history of previous coronary stent placement or coronary artery bypass grafting, the presence of metal fixation devices at the L4 level or missing essential data within one month of the coronary CTA procedure. Missing data points included: fasting plasma glucose (glucose (AC)), hemoglobin A1c (HbA1c), high-density lipoprotein (HDL), low-density lipoprotein (LDL), total cholesterol, triglycerides (TG), anthropometric data (i.e., weight, height, WC), or diastolic and systolic blood pressure (DBP and SBP) readings.

### 2.2. Ethical Considerations

The protocol for this retrospective study was approved by the local institutional review board (IRB) of Chi Mei Medical Center. The study was conducted in accordance with the principles outlined in the Declaration of Helsinki. The requirement for obtaining informed consent from included patients was waived by the IRB because the images were de-identified and patients remained anonymous.

### 2.3. Anthropometry and Laboratory Tests

Height, weight, and WC were assessed with patients maintained in the standing position. Body mass index (BMI) was calculated as body weight in kilograms divided by height in meters squared (kg/m^2^). Per established criteria [23,24], a BMI of 27 kg/m^2^ or higher, or a WC exceeding 80 cm for women and 90 cm for men was indicative of abdominal obesity and elevated health risk.

DBP and SBP were measured following a minimum rest period of 10 min. A history of hypertension was recorded, with current hypertension defined as an SBP of 130 mm Hg or higher, or a DBP of 80 mm Hg or higher.

Venous blood samples were collected after an overnight fast (minimum of 8 h) to measure fasting glucose (AC), hemoglobin A1c (HbA1c), high-density lipoprotein (HDL), low-density lipoprotein (LDL), total cholesterol, and triglycerides (TG). Diabetes mellitus (DM) was diagnosed if the fasting glucose (AC) was 126 mg/dL or higher, or the HbA1c value was 6.5% or higher; a pre-existing history of DM was also recorded. Hyperlipidemia was determined by the presence of any of the following criteria: a total cholesterol of 200 mg/dL or higher, TG of 200 mg/dL or higher, LDL of 130 mg/dL or higher, or HDL of 40 mg/dL or lower.

### 2.4. CT Image Acquisition

All CT examinations were performed using one of three multi-detector CT scanners: Somatom Definition Flash (Siemens, Munich, Germany); Somatom Definition Force (Siemens, Munich, Germany); and Brilliance, iCT 256 (Philips Healthcare, Amsterdam, The Netherlands). All abdominal CT scans were executed using a helical scan protocol, and the axial images were reconstructed with a slice thickness of 5.0 mm sections at 0.0 mm intervals. Coronary CTA examinations were employed either a sequential scan (heart rate ≤ 70 beats/min) or a helical scan (heart rate > 70 beats/min) coordinated with electrocardiographic gating and tube voltage (100 or 120 kV), tube current, and the volume of iodine-based contrast medium administered were adjusted based on the patient’s BMI. A standard soft-tissue kernel was consistently applied for all image reconstructions. Sublingual glyceryl trinitrate was administered to patients unless a specific contraindication was present.

### 2.5. Abdominal CT Image Assessment

Before being reviewed, all images were randomly assigned a new identification number, and the original identification numbers, patients’ names, and study descriptions were deleted by a radiologist with 30 years of experience. Axial images at the level of L4 pedicles were selected for further imaging analysis by the same radiologist.

Valuable pixels from the image data were extracted from reconstructed DICOM files using MATLAB (R2023a, The MathWorks, Inc., Natick, MA, USA). Pixels within the −205 to −50 HU range were selected for fat tissue examination, and pixels within the 20 to 50 HU range were selected for muscle tissue [13]. Gray-scale images were then formed with the pixels selected above, and annotated for VAT, SAT, and paraspinal muscles (PsMs), and then measured using MATLAB. Measurements were performed three times, using the average for further statistical analysis. Intra-reader reproducibility was evaluated using the intraclass correlation coefficient based on a two-way mixed-effects model with absolute agreement.

### 2.6. Coronary CTA Image Assessment

Coronary artery stenosis and the presence of plaques were rigorously evaluated using commercially available cardiac reconstruction software packages, specifically Aquarius iNtuition (TeraRecon, Foster City, CA, USA), Syngo via VB60A (Siemens, Munich, Germany), or Vitrea (Vital Images Inc., Toshiba Medical, Minnetonka, MN, USA). Assessment was performed on both axial and curved multiplanar reformatted (MPR) images. A significant coronary artery stenosis was defined as any luminal narrowing exceeding 50% of the vessel diameter, as measured consistently across both straight and curved multiplanar reconstruction images derived from the coronary CTA data [25].

### 2.7. Statistical Analysis

All statistical analyses were conducted using SAS version 9.4 (SAS Institute, Inc., Cary, NC, USA). Categorical variables are presented as frequencies with corresponding percentages. Differences in distribution between groups with and without significant coronary artery stenosis were assessed using Pearson’s Chi-square test. Continuous variables are expressed as the mean with the standard deviation (SD). The normality of these continuous variables was evaluated using both the Kolmogorov–Smirnov test and Quantile-Quantile plots; all variables were determined to follow an approximately normal distribution. Accordingly, comparisons between the groups were performed using Student’s *t*-test.

The receiver operating characteristic (ROC) curve was used to evaluate diagnostic test performance, and the area under the ROC curve (AUC) was calculated. Logistic regression was used to estimate the association between VAT and the presence of significant coronary artery stenosis. Both crude and adjusted odds ratios (ORs) are reported with 95% confidence intervals (CIs). The adjusted odds ratios were estimated using multivariable logistic regression, controlling for potential confounding factors identified as significant in the initial univariate analysis.

## 3. Results

**Figure 1:**
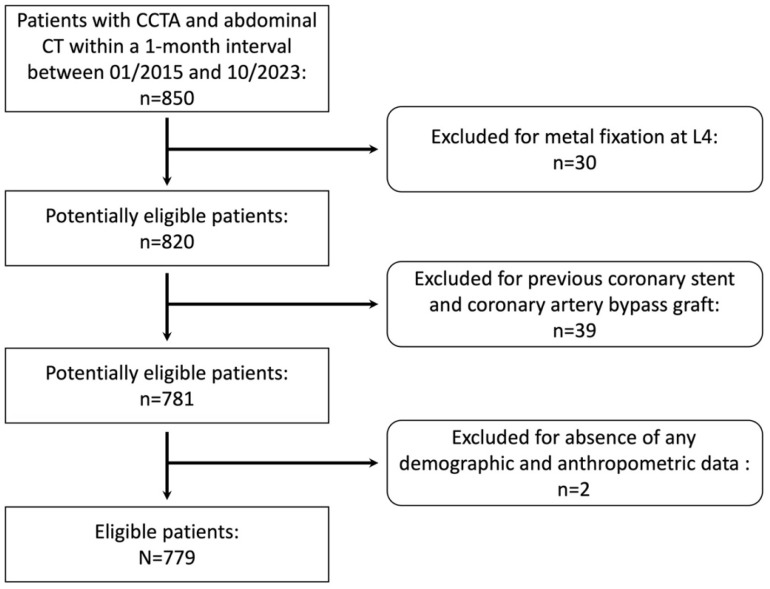
Study population selection.

Of 850 patients who initially met the inclusion criteria, 779 (300 females; mean age, 54.9 ± 9.96 years; range, 24–84 years) were ultimately enrolled after exclusions. Figure 1 presents the flowchart of study population selection based on inclusion and exclusion criteria.

Table 1 depicts the demographic and anthropometric characteristics of the study participants. Males with significant coronary artery stenosis had a significantly higher percentage than those without (84.85% vs. 56.72%, *p* < 0.0001). However, only 20 females had significant coronary artery stenosis. The mean age of the group with significant coronary artery stenosis was significantly higher than that of the group without significant coronary artery stenosis (61.39 ± 8.23 years vs. 53.55 ± 9.76, *p* < 0.001). No significant differences were found in other demographic and anthropometric characteristics between the groups with and without significant coronary artery stenosis (Table 1).

Table 2 presents dichotomous data on the demographic and anthropometric characteristics of the study subjects. The cut-off points for these characteristics are based on the official standard of Taiwan. When waist circumference was dichotomized using sex-specific cut-off values, a significant difference in the distribution of high waist circumference was observed between patients with and without significant coronary artery stenosis in the overall cohort (*p* = 0.0141). However, this difference was no longer evident after stratification by sex. Additionally, no significant differences were observed in the other dichotomized variables (Table 2).

Table 3 shows the imaging data, including VAT, SAT, and PsMs. Only VAT of the group with significant coronary artery stenosis was significantly higher than in those without significant coronary artery stenosis (150.73 ± 78.17 vs. 114.24 ± 63.21, *p* < 0.0001). The intra-reader reproducibility of VAT, SAT, and PsM measurements was excellent, with an intraclass correlation coefficient of 0.9991 (95% CI: 0.9990–0.9992), 0.9994 (95% CI: 0.9993–0.9995), and 0.9453 (95% CI: 0.9386- 0.9514), respectively.

**Figure 2:**
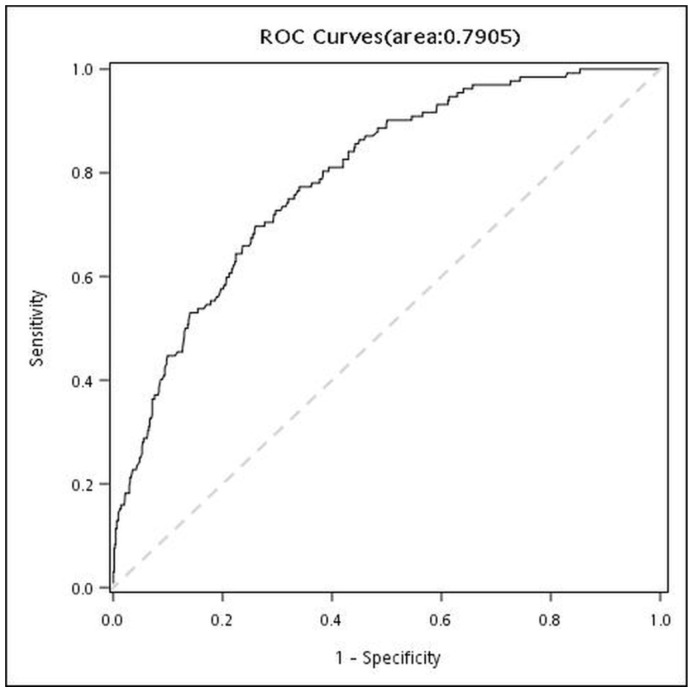
Receiver-operating characteristics (ROC) curves for cut-off values of VAT associated with CAD.

Figure 2 is the ROC curve of VAT. The cut-off value of 86.8 was recommended, and the AUC was 0.7905. Table 4 presents the results of univariate and multivariable logistic regression analysis for examining factors related to CAD. After adjustment for age, sex, BMI, high WC, hypertension, hyperlipidemia, and DM, VAT was significantly associated with CAD (adjusted OR: 2.51, 95% CI: 1.39–4.54, *p* = 0.0022).

## 4. Discussion

Based on our hospital-based data, elevated VAT values remained significantly associated with significant coronary artery stenosis even after adjustment for potential confounding factors. In contrast, other CT-derived parameters, such as SAT and PsM measurements, as well as clinical factors including BMI, hypertension, hyperlipidemia, and DM, were not significantly associated with the presence of significant coronary artery stenosis in this cohort. These findings strongly suggest that elevated VAT functions as an independent cardiometabolic risk factor, thereby supporting the use of a specific VAT cut-off value of 86.8 cm^2^ to predict the risk of significant coronary artery stenosis.

Previous research has already established that VAT is linked not only to multiple metabolic risk factors for CAD but also to adverse coronary plaque morphology and characteristics [15,26,27,28,29,30,31,32,33,34,35]. CT-based VAT quantification methodologies typically include volumetric and cross-sectional area measurements, both of which consistently demonstrate strong associations with CAD risk [29,30,31,32,33,34,35]. In the current study, a single-slice area measurement on an axial CT image was employed due to its inherent simplicity, high reproducibility, and practical applicability in routine clinical settings. Consistent with prior studies, our findings reconfirm that elevated VAT values are significantly associated with significant coronary artery stenosis. The high odds ratio observed further substantiates that single-slice CT-measured VAT is a viable and clinically useful method for predicting significant coronary artery stenosis.

The optimal VAT cut-off value identified in this study was 86.8 cm^2^, which demonstrated robust diagnostic performance. In comparison, earlier studies frequently reported higher thresholds, typically around 100 cm^2^, for predicting CAD, metabolic syndrome, or related morbidities [9,20,22,31]. However, more recent research has proposed lower cut-off values, specifically in the 80–85 cm^2^ range [9,22], which are consistent with our current findings. The observed decline in general VAT thresholds over time may reflect evolving lifestyle patterns, improved medical management strategies, and effective preventive interventions. Future large-scale, multiethnic studies incorporating detailed lifestyle and pharmacological data are warranted to refine population-specific VAT cut-offs and improve the accuracy of subsequent risk stratification.

Unlike VAT, SAT demonstrated no significant difference between patients with and without significant coronary artery stenosis in our cohort. In the seminal Framingham Heart Study, both VAT and SAT initially correlated with cardiovascular risk factors [15]. However, subsequent evidence consistently indicates that only VAT, rather than SAT, is independently associated with CAD [26,31]. This alignment with our observations reinforces the distinct roles of different fat depots. The weaker association between SAT and CAD may be biologically explained by SAT containing fewer inflammatory and immune cells and exhibiting lower metabolic activity than VAT [15].

WC is considered the most straightforward and direct anthropometric method for identifying abdominal obesity, which is robustly associated with an increased risk of CAD [36,37,38]. However, optimal cut-off points vary across different sexes and ethnicities [39,40,41,42,43]. The results of the present study indicated a significant difference in categorical WC data between groups with and without significant coronary artery stenosis, though this difference was not observed in continuous WC data. Furthermore, this association was no longer evident after stratification by sex. Although increased WC is generally associated with increased CAD risk, a previous meta-analysis demonstrated only satisfactory diagnostic performance (pooled AUC: 0.69 in men and women) [36]. Elevated WC is primarily a result of increased adipose tissue, encompassing both visceral (VAT) and subcutaneous (SAT) fat; the substantial contribution of SAT may consequently reduce the overall diagnostic performance of WC alone. The visceral adiposity index, which integrates WC, BMI, TG, and HDL-C measurements, may offer more accurate insights into visceral fat function for comprehensive cardiometabolic risk assessment [4,44].

While hypertension, hyperlipidemia, and DM are universally recognized as established risk factors for CAD [2], these conditions did not differ significantly between the CAD and non-CAD groups in our specific analysis. This observation may be ascribed to the limited sample size and, more importantly, to the widespread adoption of early pharmacologic interventions facilitated by Taiwan’s National Health Insurance system. This system enables the early detection and effective management of chronic diseases. The timely treatment of these conditions may thus attenuate their apparent statistical association with CAD within observational datasets.

Several limitations of the present study should be acknowledged. First, this investigation employed a single-center retrospective cross-sectional study design. This approach inherently introduces potential selection bias and restricts the generalizability of the findings to broader populations. Second, the study cohort comprised cases that had undergone abdominal CT within one month of their coronary CTA. The primary objective was to minimize variations in patient condition between the two examinations. While these strict inclusion criteria successfully minimized temporal variation, they simultaneously resulted in a highly selected, high-risk imaging population rather than a general or screening population. Third, the relatively small sample size, particularly among female participants, constrained the analysis’s statistical power and limited its external validity. Fourth, essential data regarding medication use (e.g., lipid-lowering, antidiabetic, or blood pressure-lowering agents, including Glucagon-Like Peptide-1), smoking history, and lifestyle behaviors (e.g., diet and physical activity) were unavailable. This absence of data represents a potential confounder of the observed associations; further studies incorporating a complete medication history are warranted to provide more granular information. Fifth, the outcome of this study is angiographic presence of significant stenosis, not clinical events. This study aims to assess the ability of VAT to screen for coronary stenosis, and patients requiring further evaluation and treatment (stenosis >50%) were selected as the study target. Finally, the inherent cross-sectional design precludes establishing causal inferences regarding the association between VAT and significant coronary artery stenosis.

In conclusion, abdominal obesity, particularly VAT, was independently and significantly associated with the presence of significant coronary artery stenosis, whereas SAT, WC, and BMI were not. Single-slice CT-measured VAT provides a reliable and practical indicator for the risk assessment of significant coronary artery stenosis, and a lower VAT cut-off value (approximately 86.8 cm^2^) is recommended for the Taiwanese population.

## Figures and Tables

**Table 1:** Demographic and anthropometric characteristics of the study subjects.

	Overall (*n* = 779)	Without CAD (*n* = 647)	with CAD (*n* = 132)	*p*-Value
Sex				<0.0001
Female, *n* (%)	300 (38.51)	280 (43.28)	20 (15.15)	
Male, *n* (%)	479 (61.49)	367 (56.72)	112 (84.85)	
Age (years)	54.88 ± 9.96	53.55 ± 9.76	61.39 ± 8.23	<0.0001
Height (cm)	164.93 ± 8.22	164.81 ± 8.27	165.54 ± 7.95	0.3484
Weight (kg)	67.07 ± 12.74	66.96 ± 12.66	67.64 ± 13.17	0.5758
BMI (kg/m^2^)	24.53 ± 3.54	24.53 ± 3.55	24.54 ± 3.54	0.9815
WC (cm)	83.03 ± 10.13	82.84 ± 10.04	83.97 ± 10.53	0.2426
Female	80.94 ± 9.80	80.93 ± 9.81	81.08 ± 9.92	0.9503
Male	84.33 ± 10.12	84.29 ± 9.98	84.48 ± 10.59	0.8642
SBP (mmHg)	119.92 ± 16.99	119.32 ± 17.01	122.84 ± 16.65	0.0287
DBP (mmHg)	75.70 ± 11.76	75.40 ± 11.90	77.19 ± 10.99	0.0943
HbA1c (%)	5.95 ± 0.92	5.94 ± 0.91	6.01 ± 0.96	0.4397
Glucose (AC) (mg/dL)	103.85 ± 28.28	103.44 ± 27.53	105.85 ± 31.73	0.4181
Total Cholesterol. (mg/dL)	203.03 ± 45.80	202.38 ± 42.11	206.26 ± 60.83	0.4851
LDL (mg/dL)	129.04 ± 38.64	128.91 ± 37.82	129.64 ± 42.61	0.8448
HDL (mg/dL)	47.10 ± 13.14	47.29 ± 13.33	46.17 ± 12.14	0.3700
TG (mg/dL)	147.60 ± 109.86	144.66 ± 90.23	162.01 ± 176.84	0.2739
Comorbidity				
Hypertension, *n* (%)	300 (38.51)	241 (37.25)	59 (44.70)	0.1090
Hyperlipidemia, *n* (%)	618 (79.33)	509 (78.67)	109 (82.58)	0.3126
DM, *n* (%)	130 (16.69)	106 (16.38)	24 (18.18)	0.6135

Notes: Data are expressed as mean ± standard deviation (SD) unless otherwise indicated. Abbreviations: CAD, Coronary artery disease; BMI, body mass index; WC, waist circumference; SBP, systolic blood pressure; DBP, diastolic blood pressure; HbA1c, hemoglobin A1c; Glucose (AC): fasting plasma glucose; LDL: low-density lipoprotein; HDL: high-density lipoprotein; TG: triglyceride; DM: diabetes mellitus.

**Table 2:** Influence of the cut-off points of potential risk factors on coronary arterial stenosis.

*n* (%)	Overall (*n* = 779)	Without CAD (*n* = 647)	with CAD (*n* = 132)	*p*-Value
BMI (kg/m^2^)				0.6472
BMI < 27	602 (77.28)	502 (77.59)	100 (75.76)	
BMI ≥ 27	177 (22.72)	145 (22.41)	32 (24.24)	
WC (cm)				0.0141
Normal WC (M < 90; F < 80)	481 (61.75)	387 (59.81)	94 (71.21)	
High WC (M ≥ 90; F ≥ 80)	298 (38.25)	260 (40.19)	38 (28.79)	
Female WC (cm)				0.4581
Normal WC (<80)	141 (47.00)	130 (46.43)	11 (55.00)	
High WC (≥80)	159 (53.00)	150 (53.57)	9 (45.00)	
Male WC (cm)				0.4050
Normal WC (<90)	340 (70.98)	257 (70.03)	83 (74.11)	
High WC (≥90)	139 (29.02)	110 (29.97)	29 (25.89)	
SBP (mmHg)				0.1352
SBP ≤ 130	583 (74.84)	491 (75.89)	92 (69.70)	
SBP > 130	196 (25.16)	156 (24.11)	40 (30.30)	
DBP (mmHg)				0.2487
DBP ≤ 80	529 (67.91)	445 (68.78)	84 (63.64)	
DBP > 80	250 (32.09)	202 (31.22)	48 (36.36)	
HbA1c (%)				0.9265
HbA1c < 6.5	663 (85.11)	551 (85.16)	112 (84.85)	
HbA1c ≥ 6.5	116 (14.89)	96 (14.84)	20 (15.15)	
Glucose (AC) (mg/dL)				0.2116
Glucose (AC) < 126	686 (88.06)	574 (88.72)	112 (84.85)	
Glucose (AC) ≥ 126	93 (11.94)	73 (11.28)	20 (15.15)	
Total Cholesterol (mg/dL)				0.2737
Total Cholesterol < 200	382 (49.04)	323 (49.92)	59 (44.70)	
Total Cholesterol ≥ 200	397 (50.96)	324 (50.08)	73 (55.30)	
LDL (mg/dL)				0.4809
LDL < 130	423 (54.30)	355 (54.87)	68 (51.52)	
LDL ≥ 130	356 (45.70)	292 (45.13)	64 (48.48)	
HDL (mg/dL)				0.6994
Normal HDL (M > 40; F > 50)	413 (53.02)	341 (52.70)	72 (54.55)	
Low HDL (M ≤ 40; F ≤ 50)	366 (46.98)	306 (47.30)	60 (45.45)	
TG (mg/dL)				0.3180
TG < 200	632 (81.13)	529 (81.76)	103 (78.03)	
TG ≥ 200	147 (18.87)	118 (18.24)	29 (21.97)	

Abbreviations: CAD, Coronary artery disease; BMI, body mass index; WC, waist circumference; SBP, Systolic blood pressure; DBP, Diastolic blood pressure; HbA1c, Hemoglobin A1c; Glucose (AC), fasting plasma glucose; LDL: Low-density lipoprotein; HDL: High-density lipoprotein; TG: Triglyceride.

**Table 3:** CT data of the study subjects.

	Overall (*n* = 779)	Without CAD (*n* = 647)	with CAD (*n* = 132)	*p*-Value
VAT (cm^2^)	120.42 ± 67.34	114.24 ± 63.21	150.73 ± 78.17	<0.0001
SAT (cm^2^)	173.32 ± 75.13	174.16 ± 76.25	169.21 ± 69.54	0.4643
PsMs (cm^2^)	18.25 ± 4.05	18.21 ± 4.04	18.46 ± 4.09	0.5283

Note: Values in the table are expressed as mean ± SD. Abbreviations: CAD, Coronary artery disease; VAT, visceral adipose tissue; SAT, subcutaneous adipose tissue; PsMs, paraspinal muscles.

**Table 4:** Factors associated with CAD: multivariable logistic regression analysis.

Factor	Crude OR (95% CI)	*p*-Value	Adjusted OR (95% CI)	*p*-Value
High VAT (>86.8)	4.28 (2.48,7.38)	<0.0001	2.51 (1.39,4.54)	0.0022
Male sex	4.27 (2.59,7.05)	<0.0001	3.19 (1.82,5.60)	<0.0001
Age	1.10 (1.07,1.13)	<0.0001	1.10 (1.07,1.12)	<0.0001
High BMI (≥27)	1.11 (0.71,1.72)	0.6473	1.25 (0.69,2.27)	0.4563
High WC	0.60 (0.40,0.91)	0.0147	0.66 (0.38,1.67)	0.1530
Hypertension	1.36 (0.93,1.99)	0.1098	1.18 (0.78,1.79)	0.4406
Hyperlipidemia	1.29 (0.79,2.09)	0.3137	1.49 (0.87,2.55)	0.1432
DM	1.13 (0.70,1.85)	0.6137	0.97 (0.56,1.68)	0.9209

Note: Multivariable logistic regression model adjusted for age, sex, BMI, WC, hypertension, hyperlipidemia, and DM. Abbreviations: CAD, Coronary artery disease; VAT, visceral adipose tissue; OR, odds ratio; BMI, Body mass index; WC, Waist circumference; DM, Diabetes mellitus.

## Data Availability

No new data were created or analyzed in this study. Data sharing is not applicable to this article.

## Abbreviations

The following abbreviations are used in this manuscript:

CT	Computed tomography
CTA	Computed tomography angiography
CAD	Coronary artery disease
BMI	Body mass index
WC	Waist circumference
SBP	Systolic blood pressure
DBP	Diastolic blood pressure
HbA1c	Hemoglobin A1c
Glucose (AC)	Fasting plasma glucose
LDL	Low-density lipoprotein
HDL	High-density lipoprotein
TG	Triglyceride
DM	Diabetes mellitus
VAT	Visceral adipose tissue
SAT	Subcutaneous adipose tissue
PsM	Paraspinal muscle
OR	Odds ratio
